# Assembly-free typing of Nanopore and Illumina data through proximity scoring with KMA

**DOI:** 10.1093/nargab/lqaf116

**Published:** 2025-09-01

**Authors:** Philip T L C Clausen, Malte B Hallgren, Søren Overballe-Petersen, Vanessa R Marcelino, Henrik Hasman, Frank M Aarestrup

**Affiliations:** Research Group for Genomic Epidemiology, National Food Institute, Technical University of Denmark, 2800 Kgs. Lyngby, Denmark; Research Group for Genomic Epidemiology, National Food Institute, Technical University of Denmark, 2800 Kgs. Lyngby, Denmark; Department of Bacteria, Parasites, and Fungi, Statens Serum Institut, 2300 Copenhagen, Denmark; Department of Microbiology and Immunology at The Peter Doherty Institute, University of Melbourne, VIC 3010, Australia; Melbourne Integrative Genomics, School of BioSciences, University of Melbourne, Parkville, VIC 3010, Australia; Department of Bacteria, Parasites, and Fungi, Statens Serum Institut, 2300 Copenhagen, Denmark; Research Group for Genomic Epidemiology, National Food Institute, Technical University of Denmark, 2800 Kgs. Lyngby, Denmark

## Abstract

Advances in Oxford Nanopore Technologies (ONT) with the introduction of the r10.4.1 flow cell have reduced the sequencing error rates to <1%. When a reference sequence is known, this allows for accurate variant calling comparable with what is known from the second-generation short-read sequencing technologies, such as Illumina. Additionally, the longer sequence reads provided by ONT enable more efficient mappings, which means the amount of multimapping reads is reduced. However, when the correct reference is not known in advance, and the target reference is highly similar to other references, the multimapping problem is still a concern. Although the *ConClave* algorithm has provided an accurate solution to the multimapping problem of the second-generation short-read sequencing technologies, it is less effective when resolving the multimapping problems arising from third-generation long-read sequencing technologies. To overcome this problem, we are introducing proximity scoring of alleles, which aids the *ConClave* algorithm to accurately assign specific alleles from databases containing loci with a high degree of redundancy. Using multilocus sequence typing as a test case, we show that this approach matches the results obtained from sequencing data of Illumina while using limited computational resources that essentially correspond to that of today’s smartphones.

## Introduction

Over the past decade the second-generation (short-read) sequencing technologies have ensured gradually cheaper and faster taxonomic identification and typing of microorganisms compared with traditional molecular typing methods [1–3]. With the introduction of the third-generation (long-read) sequencing technologies, Oxford Nanopore Technologies (ONT) offers sequencing reagents at a price range matching that of Illumina (short-read sequencing) and significantly more affordable sequencing equipment. Thus, sequencing is no longer limited to specialized centers and laboratories but can be initialized locally in small laboratories and research groups, allowing for a larger inclusion of low- and middle-income countries [4]. However, computational infrastructure to process the vast amount of data produced by these third-generation sequencers remains a major impediment for many laboratories [5].

For analyses of second-generation sequence data, several solutions that do not require more computational power than what is provided through a laptop computer with 8–16 gigabytes (GB) of memory have been developed [5–7]. This has been achieved through methods that do not rely on *de novo* assembly, which is a time-consuming process that would have left out laboratories without access to high-performance computing [5, 7, 8]. Recently, Hallgren *et al.* 2021 showed that single nucleotide polymorphism (SNP) level annotation could be carried out on conventional laptop computers using both second- and third-generation sequencing data and even provide results that are independent of different versions of ONT sequencing [9].

A key characteristic of the third generation sequence data is that the generated sequences are longer but more prone to errors. This makes mapping of sequences easier, as longer stretches of DNA usually lead to more confident mappings [10]. However, molecular typing still remains a problem when analyzing third-generation sequence data, as the error rate is higher than the resolution of the target sequences [7]. When performing multilocus sequence typing (MLST), the reference sequences often differ by just a few SNPs, which in turn causes errors that match closely related alleles better than the actual reference. A similar problem is observed with the second-generation sequence data, where short reads do not always span regions of variation between references, giving rise to the multimapping problem [7, 11, 12]. The ability to distinguish between highly similar references is a key feature when performing microbial typing, as even single SNPs might lead to alternative phenotypes and alternative sequence types [7]. We previously developed a *k*-mer alignment (KMA) method that allows for direct alignment of raw reads against entire databases without the need of similarity reduction [7]. KMA uses an extra mapping step where the reference of each input sequence is found and scored with the *ConClave* algorithm. KMA has, since the publication of the first version, undergone several improvements and is today widely used, with >700 citations spanning several fields of research and has >216K downloads at Bioconda (19/02/2025), putting it in the top 2.5% of available software at BioConda [13].

To solve the problem of molecular typing of third-generation sequence data, we here present an updated version (>1.4.0) of KMA. The updated version of KMA enables direct typing of assembly-free raw sequencing data (fastq) from both second- and third-generation sequence data, using sequence databases with a high amount of redundant sequence patterns, e.g. MLST. Here we present proximity scoring as a solution to accurately place raw sequencing where the error rate exceeds that of the database resolution.

## Materials and methods

KMA utilizes a two-step mapping approach prior to alignment, which, combined with the *ConClave* algorithm, has proven useful when mapping and aligning sets of sequences with a high degree of redundant sequence patterns [7, 14–17].

Prior to alignment, KMA requires an indexed database of target references, where a HashMap is created with the key being the *k-*mer and the value a set of reference sequence identifiers (denoted *k*-mer signatures), used for the first mapping. This indexing falls between that of a traditional aligner, such as minimap2 or BLAST, where the position of each *k*-mer is stored [18, 19], and strict mapping-based methods, such as Kraken or Kraken2, where the lowest common ancestor is stored [20, 21]. Only saving the reference sequence identifiers for each *k*-mer allows for a more efficient compression of the final index, as large sets of similar sequences will share *k*-mers and result in redundant signatures that can be collapsed. When mapping sequences using *k-*mer signatures, some specificity is lost when compared to traditional aligners. To adjust for the lost specificity, KMA creates a HashMap for matched target references at runtime, which stores the *k-*mer positions for matched target references only. This way the first index is used to get a set of target references that is likely to match, while the second index is used to verify these references by performing an additional within-reference mapping. The latter mapping step follows a typical seed-chain-align procedure, where matched *k-*mers are extended, chained together, and connected with dynamic programming [19].

Multi-mapping query sequences are then resolved using the *ConClave* algorithm (described later), and a consensus sequence is generated for each matched reference sequence by creating a pileup of alignments while aligning the sequences as described in Clausen *et al.* 2018 and Hallgren *et al.* 2021.

### Identification of reference candidates

As in the original version of KMA, *k*-mer signatures are stored in a HashMap, which is used to map a query sequence against a set of reference sequences. Only saving the information of presence for each *k*-mer allows for a more efficient compression of the final index, as large sets of similar sequences will share *k*-mers and result in redundant signatures that can be collapsed.

Given a list of maximal exact matches (MEMs or anchors) of *k-*mer signatures between a query sequence and a database, let *f*(*i*) be the maximum chaining score of each anchor (*i*).

Then, by maximizing collinearity between database and query, *f*(*i*) can be calculated dynamically as:


(1) \begin{eqnarray*}
f\left( i \right) = {{w}_i} + {\mathrm{max}} \left\{ {\mathop {\max }\limits_{1 \le j \le i} \left\{ {f\left( j \right) + p\left( {i,j} \right)} \right\},{\mathrm{\ }}o\left( i \right)} \right\}.
\end{eqnarray*}

Where *w_i_* is the weight of anchor *i*, length of anchor times reward for matching bases, *p*(*i*,*j*) is the cost of extending the chain from anchor *i* to anchor *j*, and *o*(*j*) is cost of starting a new chain at anchor *i*.

The cost of extending the chain, *p*(*i*,*j*), depends on the overlap between anchors *i* and *j, g_i,j_*, where a perfect overlap (*k −1*) is penalized by subtracting the matching bases between them, as these are already accounted for with *w_i_* in (Equation 1). Other overlaps by anchors indicates a deletion in the query sequence and is penalized as such, while non-overlapping anchors are penalized with the minimal number of insertions and mismatches needed to chain them together. The extension cost, *p*(*i*,*j*), is calculates as:


(2) \begin{eqnarray*}
p\left( {i,j} \right) = \left\{ {\begin{array}{@{}*{1}{c}@{}} {\left( {1 - k} \right)\ M\ {{g}_{i,j}} = 1 - k}\\ {{{g}_{i,j}}\ M + \left( {\left| {{{g}_{i,j}}} \right| - 1} \right)U + W\ {{g}_{i,j}} < 0}\\ {{\rm min}\left\{ {\begin{array}{@{}*{1}{c}@{}} {\lceil \frac{{{{g}_{i,j}}}}{k}\rceil \ E + \min \left\{ {{{g}_{i,j}} - \lceil \frac{{{{g}_{i,j}}}}{k}\rceil , \frac{{{{g}_{i,j}}}}{k}\rceil ,{\mathrm{\ }}k} \right\}M}\\ {W + \left( {{{g}_{i,j}} - 1} \right)\ U} \end{array}} \right.\ {\rm else}} \end{array}} \right..\nonumber\\
\end{eqnarray*}

Where *k* is the *k-*mer size, *M* are reward for a matching base and *E, W* and *U* are the penalties for a mismatch, gap opening and gap extension, respectively.

When extending a chain is not feasible, e.g. when a reference is completely contained in a query sequence, a chain opening cost, *o*(*i,j*), is added to the weight of the anchor instead. This is zero if the space between anchor *i* and the end of the query sequence, *gĝ_i_*, is zero, elsewhere a gap cost is added between anchor *i* and the end of query sequence to max penalty of *L*. The opening cost, *o*(*i,j*), is calculated as:


(3) \begin{eqnarray*}
o\left( i \right) = \left\{ {\begin{array}{@{}*{1}{c}@{}} 0\\ {{\rm max}\left\{ {L,W + \left( {{{{\hat{g}}}_i} - 1} \right)U} \right\}} \end{array}{\mathrm{\ }}\begin{array}{@{}*{1}{c}@{}} {{{{\hat{g}}}_i} = 0}\\ {{\rm else}} \end{array}} \right..
\end{eqnarray*}

(Equation 3) is applied to each chain to penalize the opening space towards the beginning of the query, and chains exceeding a user-defined threshold is saved as valid chains.

Overlapping chains are discarded by sorting the valid chains according to their score, and adding them in descending order to a segment-tree, which determines their overlap towards accepted chains.

Reference candidates of each query sequence can then be extracted by backtracking the chain with highest score through (Equation 1), i.e. the reference candidates will be the ones satisfying *argmax* of (Equation 1).

### Pairwise sequence alignment

Query sequences with assigned reference candidates are re-mapped to identify MEMs (anchors) between single query and reference sequences, where maximal co-linear *k*-mer matches are extended prior to chaining using (Equation 4). Which is inspired by the chaining performed with minimap2 [19], but refined to fit global alignments. Then the maximum chain, *f*(*i*), for each MEM can be calculated as:


(4) \begin{eqnarray*}
f( i ) = {{w}_i} + {\mathrm{max}} \left\{ {\mathop {\max }\limits_{1 \le j \le i} \left\{ {f( j ) + \alpha ( {i,j} ) + \beta ( {i,j} )} \right\},{\mathrm{\ \gamma }}( {\mathrm{i}} )} \right\}.\nonumber\\
\end{eqnarray*}

Where *w_i_* is the weight of anchor *i*, like in (Equation 1), *α*(*i,j*) estimates the cost of joining anchor *i* with anchor *j* with respect to (mis)matches, *β*(*i,j*) estimates the cost of joining anchor *i* with anchor *j* with respect to gaps, and *γ*(*i*) estimates the cost of creating a new chain from anchor *i*.

The estimate of (mis)matching bases between two anchors, *α(i,j)*, is zero when the distance between the anchors, *m_i,j_*, is zero on either the query or reference sequence. Otherwise at least one mismatch must be encountered per *k-*mer between the anchors, which is relaxed with a with the same amount of matches up to a max of *k*. Where *α*(*i,j*) is calculated as:


(5) \begin{eqnarray*}
\alpha \left( {i,j} \right) = \left\{ {\begin{array}{@{}*{2}{c}@{}} 0&{{{m}_{i,j}} = 0}\\ {{\mathrm{max}}\left\{ {\lceil \frac{{{{m}_{i,j}}}}{k}\rceil ,2} \right\}{\mathrm{\ }}E + \min \left\{ {{{m}_{i,j}} - \lceil \frac{{{{m}_{i,j}}}}{k}\rceil , \frac{{{{m}_{i,j}}}}{k}\rceil ,{\mathrm{\ }}k} \right\}M}&{{\rm else}} \end{array}} \right..\nonumber\\
\end{eqnarray*}

The estimate of gaps between anchors, *β*(*i,j*), are zero when the difference in space between anchors *i* and *j, g_i,j_*, are zero with respect to both query and reference sequence. Otherwise, the chaining between the anchors is penalized with a single gap, while rewards for matching bases are subtracted if the anchors overlap. Where *β*(*i,j*) is calculated as:


(6) \begin{eqnarray*}
\beta \left( {i,j} \right) = \left\{ {\begin{array}{@{}*{1}{c}@{}} {\begin{array}{@{}*{1}{c}@{}} 0\\ {W + \left( {{{g}_{i,j}} - 1} \right)U} \end{array}\ }\\ {W + \left( {\left| {{{g}_{i,j}}} \right| - 1} \right)U + {{g}_{i,j}}M} \end{array}{\mathrm{\ }}\begin{array}{@{}*{1}{c}@{}} {{{g}_{i,j}} = 0}\\ {{{g}_{i,j}} > 0}\\ {{\rm else}} \end{array}} \right..
\end{eqnarray*}

The penalty of a new chain, *γ*(*i*), is zero when the distance from the end of anchor *i* to the end of the reference sequence, *gĝ_i_*, is zero. elsewhere the number of (mis)matches and gaps are estimated as in (Equations 5 and 6). The cost of starting a new chain, *γ*(*i*), is calculated as:


(7) \begin{eqnarray*}
\gamma \left( i \right) = {\rm max}\left\{ {\begin{array}{@{}*{1}{c}@{}} {w1 + \left( {{{{\hat{g}}}_i} - 1} \right)U}\\ {\lceil \frac{{{{{\hat{g}}}_i}}}{k}\rceil \ E + \min \left\{ {{{{\hat{g}}}_i} - \lceil \frac{{{{{\hat{g}}}_i}}}{k}\rceil , \frac{{{{{\hat{g}}}_i}}}{k}\rceil ,{\mathrm{\ }}k} \right\}M} \end{array}} \right..
\end{eqnarray*}

When circular sequences are aligned (set through runtime options), *m_i,j_* and *g_i,j_* are allowed to span the ends of the reference sequence, allowing for alignments of circular genomes.

After the maximum chaining score is identified, the anchors are joined using dynamic programming to get the final alignment [22, 23].

### *ConClave* with proximity scoring

To resolve multimapping query sequences, KMA uses the *ConClave* algorithm as presented in Clausen *et al. 2018*, which works in three main steps, as presented below. In brief, it starts by identifying all high-scoring reference sequences for query sequence, then the *ConClave* score is computed for each reference as the sum of alignment scores from all query sequences, and lastly a reference sequence is chosen for each query sequence as the one with the highest *ConClave* score amongst the high-scoring references for that query sequence [18, 19].

Let *T*_m_(*q*) be the set of maximum scoring reference sequences, for a given query sequence *q*, according to a mapping or alignment function *f*(*q*,*t)* for all reference sequences *t* in a set of reference sequences *T*. Then let *T*_m_(*q*) be defined:


(8) \begin{eqnarray*}
{{T}_m}\left( q \right)\epsilon \mathop {{\mathrm{argmax}}}\limits_{t{\mathrm{\ }}\epsilon {\mathrm{\ }}T} \left\{ {{\mathrm{\ }}f\left( {q,t} \right){\mathrm{\ }}} \right\}.
\end{eqnarray*}

In cases where a query sequence matches several reference sequences equally well, i.e. the query sequence is multi-mapping, *T*_m_(*q*) will contain several arguments (one for each multi-mapping reference).

We then define the *ConClave* score, *C*(*t*), for each reference sequence in the database *T*, as being the sum of the maximum scores provided by *f*(*q*,*t*) for all query sequences *Q* that exceed the threshold *τ*. The *ConClave* score, *C*(*t*), is then calculated as:


(9) \begin{eqnarray*}
C(t) = \sum\nolimits_{q \in Q} {\left\{ {\begin{array}{@{}*{1}{c}@{}} {f(q,t)\tau \le f(q,t) \wedge t \in {{T}_m}(q)}\\ {0\;{\rm else}} \end{array}} \right.}.
\end{eqnarray*}

The most likely reference sequence(s), *S_q_*, can then be identified for each query sequence *q* as the reference sequence(s) contained within the set of the highest scoring *ConClave* scores, *C*(*t*), among the highest scoring reference sequences for that query sequence *T_m_*. The predicted reference sequence(s), *S_q_*, for each query sequence is identified as:


(10) \begin{eqnarray*}
{{S}_q}\epsilon\mathop {{\mathrm{argmax}}}\limits_{t{\mathrm{\ }}\epsilon{\mathrm{\ }}{{T}_m}\left( q \right)} \left\{ {{\mathrm{\ }}C\left( t \right){\mathrm{\ }}} \right\}.
\end{eqnarray*}

By applying (Equation 8), it is assumed that base-calling errors do not result in alternative reference matches giving higher scores than the true reference sequence. This assumption is justifiable when using short accurate reads, such as those produced by Illumina and Ion Torrent, but is not justifiable for long error-prone reads, such as those produced by ONT.

In order to overcome this assumption, (Equation 8) can be replaced by (Equation 11), which accounts for base-calling errors by including reference sequences with a score within a close proximity ($\epsilon$) of the best scoring reference (s) as part of *T_m_* for each query sequence. To include this proximity scoring, *T_m_* can be redefined as:


(11) \begin{eqnarray*}
{{T}_m}\left( q \right)\epsilon\mathop {{\mathrm{argmax}}}\limits_{t{\mathrm{\ }}\epsilon{\mathrm{\ }}T} \left\{ {{\mathrm{\ min}}\left\{ {\begin{array}{@{}*{1}{c}@{}} {\mathop {\max }\limits_{r{\mathrm{\ }}\epsilon{\mathrm{\ }}T} \left\{ {f\left( {q,r} \right)} \right\}}\\ {\frac{{f\left( {q,t} \right)}}{\epsilon }} \end{array}} \right\}} \right\}{\mathrm{\ }}\epsilon \epsilon\left( {0;1} \right].
\end{eqnarray*}

This allows a broader set of reference sequences as candidates for each query, while the higher scoring reference sequences will get higher ConClave scores, as the individual scores are kept for each match in (Equation 9).

### Reassigning consensus sequences

Although resolving multimapping query sequences using the *ConClave* algorithm have proven superior to other approaches [17, 24], even though the *ConClave* algorithm will occasionally misassign query sequences to a closely related reference sequence (as noted by Davies *et al.* 2023 and the developers of RGI [25]), the consensus sequence matches the correct reference sequence in most cases [17]. Thus, the produced consensus sequence can be used to reassign the query sequences to the correct reference sequences in cases where imperfect matches are detected between consensus and reference sequences. This feature has been included in this study, where the consensus sequence of imperfect reference matches was realigned with KMA to the entire collection of reference sequences to resolve possible misassignments (see Supplementary S1). This procedure has been noted as reassignment in the result section when imperfect matches between alleles and the produced consensus sequence were imperfect and the consensus was reassigned to a perfectly matching allele.

### Evaluation

We focused on evaluating the revised KMA algorithm’s ability to correctly detect MLST allele profiles because ground truth data (MLST-type) is available and can serve as a gold standard to evaluate our new method with real data. Typing of MLST alleles is expected to work similarly well compared to detection of, for example, antimicrobial resistance genes. Antimicrobial resistance, however, is a significantly more complex trait, as it can arise through point mutations in the bacterial chromosome or by acquiring resistance genes encoded in plasmids. Additionally, closely related alleles can coexist within the same cell, making their detection challenging, as short- and long-read sequencing methods often yield discrepant results [17, 26]. Taken together, this makes them less suitable than MLST to compare results between sequencing platforms.

A total of 137 bacterial isolates covering 14 species were acquired for this study, for which both Illumina and ONT (flow cells r9.4.1, r10.3, and r10.4.1) sequence data were publicly available (see Supplementary S2). Of these, five isolates were sequenced on two different ONT flow cells, r9.4.1 and r10.3, giving a total of 142 ONT sequenced samples to evaluate (see Table 1). Of the 142 samples, 81 only had Illumina sequence data publicly available, for which ONT sequence data were added during this study.

**Table 1.** Overview of sequence data; MLST scheme

Species	N	Flow cell version	Base caller and model	Laboratory	Study	MLST scheme
*Citrobacter freundii*	17	R10.4.1	Guppy-6.4.6 sup	SSI	[29]	[30]
*C. freundii*	1	R9.4.1	Guppy-6.1.5 sup	SSI	[29]	[30]
*C. freundii*	1	R10.4.1	Guppy-6.5.7 sup	SSI	[29]	[30]
*Escherichia coli*	7	R9.4.1	Guppy-5.0.16 sup	DTU	[31]^R^	[32]
*E. coli*	5	R9.4.1	N/A	DTU	[31]^R^	[32]
*E. coli*	12	R9.4.1	Guppy-3.6.0 hacm	SSI	[9]	[32]
*E. coli*	24	R9.4.1	Guppy-5.0.11 sup	SSI	[33]^R^	[32]
*E. coli*	5	R9.4.1	Guppy-4.3.4 hac	SSI	[34]	[32]
*E. coli*	5	R10.3	Guppy-5.0.11 sup	SSI	[34]	[32]
*E. coli*	2	R10.4.1	Guppy-6.4.6 sup	SSI	[29]	[32]
*Enterobacter cloacae*	2	R9.4.1	N/A	DTU	[31]^R^	[35]
*Enterococcus faecalis*	4	R9.4.1	Guppy-5.0.16 sup	DTU	[31]^R^	[36]
*Enterococcus faecium*	1	R9.4.1	Guppy-5.0.16 sup	DTU	[31]^R^	[37]
*Klebsiella oxytoca*	1	R10.3	Guppy-5.0.11 sup	SSI	[29]	[38]
*K. oxytoca*	1	R10.4.1	Dorado-7.2.13 sup	SSI	[29]	[38]
*Klebsiella pneumoniae*	10	R9.4.1	Guppy-5.0.16 sup	DTU	[31]^R^	[39]
*K. pneumoniae*	2	R9.4.1	N/A	DTU	[31]^R^	[39]
*K. pneumoniae*	15	R10.4.1	Guppy-6.4.6 sup	SSI	[29]	[39]
*K. pneumoniae*	1	R10.4.1	Guppy-6.5.7 sup	SSI	[29]	[39]
*Pseudomonas aeruginosa*	2	R9.4.1	N/A	DTU	[31]^R^	[40]
*P. aeruginosa*	1	R9.4.1	Guppy-5.0.16 sup	DTU	[31]^R^	[40]
*Staphylococcus aureus*	11	R9.4.1	Guppy-5.0.11 sup	SSI	[41]^R^	[42]
*S. aureus*	3	R9.4.1	Guppy-5.0.11 sup	DTU	[31]^R^	[42]
Others*	9	R9.4.1	N/A	DTU	[31]^R^	[30, 37, 38, 42–46]

*; Samples of Bacillus cereus, C. freundii, E. faecium, Klebsiella aerogenes, K. oxytoca, S. aureus, Staphylococcus epidermis, Staphylococcus lugdunensis, and Streptococcus pyogenes. ^R^: Data has been re-sequenced with ONT after the original study. Species denotes the species of the isolates at each row. N denotes the number of samples at each row. Flow cell version states the version of the ONT flow cell used for sequencing. Base caller and model states base caller and model used to base call the ONT data. Laboratory gives the laboratory at which the sequencing was performed. Study references the study where the sequence data of the samples was first published. MLST scheme references the MLST scheme used for samples in each row. Additional information, such as read and base counts, is available for each sample through Supplementary S2.

For ONT sequencing, DNA was extracted with Beckman Coulter’s GenFind v3 kit using a DynaMag-2 magnet. Libraries were prepared according to ONT’s Rapid Barcoding Kit 96 (SQK-RBK110.96), followed by sequencing in a MinION Mk1B with flow cell versions and base calling models according to Table 1. Sequencing adapters were removed with Porechop v0.2.3 [27, 28] and quality filtered to Q ≥ 10 with KMA v1.4.15 [7].

### Validation

The MLST schemes (see Table 1) were retrieved from pubmlst.org (19/06/2023) [38] and indexed with KMA (v1.4.15) using the -C option and a minimizer size of 14 for the ONT data [7].

The Illumina data were *de novo* assembled using SPAdes (v3.15.5) with the –only-assembler option [47], and the MLST alleles were identified in these using BLAST (v2.14.0+) through the CGE MLST service (v2.0.9) [18, 48]. Where the MLST alleles, derived from *de novo* assembled Illumina data, will serve as the ground truth and current gold standard of MLST typing when validating the identified alleles using raw sequence data from Illumina and ONT [38, 49].

To our knowledge, no other tools exist that are designed to differentiate between closely related reference sequences. However, as a baseline comparison, KMA was evaluated along with minimap2 (v2.29-r1283) [19], Winnowmap2 (v2.03) [50], and Krocus (v1.0.3) [51]. Where minimap2 is a general-purpose long-read aligner, upon which Winnowmap2 adds heightened sensitivity in repeat areas that to some extend is comparable to the multi-mapping problem presented in this study. Depth of coverage and breadth of coverage were calculated using samtools (v1.21) [52] and bedtools (v2.31.1) [53], with a mapping quality cutoff of 5 to accept a hit on a specific allele [50, 54]. Krocus is solely mapping-based and does not allow alignment, nor is it suited to other purposes than MLST. Krocus is built specifically to MLST where each allele can be assumed to belong to a single copy locus, which complicates the use for other tasks as previously shown [7, 17]. Although nanoMLST is able to provide MLST for ONT data, it is built to substitute Sanger sequencing with ONT sequencing while the rest of the MLST follows the traditional typing process with PCR amplification [55]. As nanoMLST does not support WGS data, it was not included in the comparison.

## Results

### MLST of long and short sequence data

The alleles of each locus were extracted as the one with the highest depth of coverage and a reference identity of at least 95% and counted as a match with the assembly-based typing if and only if the assigned allele had 100% breadth of coverage and 100% identity with the assigned MLST allele.

Full concordance was observed when analyzing raw Illumina data with KMA except for two misassigned alleles. Here, the same allele from two separate *Escherichia coli*isolates was misassigned; *purA_7* misassigned as *purA_489*. However, both consensus sequences were correctly reassigned as *purA_7* when realigning the consensus sequences back to the *E. coli* MLST scheme. The two misassigned alleles were sequenced to a depth of coverage of 45.30 and 64.65, whereas the rest of the Illumina samples were sequenced to a depth ranging from 4.91 to 534.48 based on the KMA output. When comparing the *purA_489* of the *E. coli* scheme with the other alleles of *purA*, it revealed an inclusion of 12 nucleotides upstream of the traditional starting position of *purA*. The inclusion of upstream DNA caused better matches when using Illumina data for some read pairs, which in turn gave slightly higher *ConClave* scores for the incorrect allele independently of the sequencing depth. Although the consensus sequences of the two misassigned samples were correct and allowed for a correct reassignment, editing the beginning of *purA_489* to match the start position of the additional alleles at the loci removed the need of reassigning the two misassigned alleles for *E. coli*. The curators of the *E. coli* MLST scheme has been informed about the apparent inclusion of upstream DNA at *purA_489*.

When analyzing ONT data with KMA, a total of 130 alleles were misassigned when proximity scoring was disabled, of which 10 were reassigned correctly. This analysis gave an accuracy of 87.93% and 86.92% with and without reassignment of imperfect matches, respectively.

**Figure 1.**
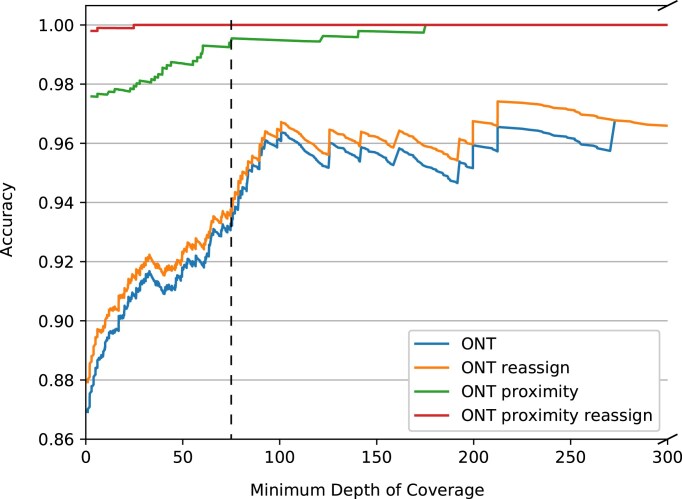
Accuracy of MLST allele assignment from ONT data when compared with *de novo* assembled Illumina data, with and without proximity scoring and reassignment. The dotted line indicates a depth of coverage of 75. Note the truncation where reassignment did not increase accuracy. Krocus was not included, as no information was available on either depth of coverage, breadth of coverage, or identity. Winnowmap2 and minimap2 spread the alignments over several alleles for each locus, for which the generation of a consensus sequence was deemed infeasible.

In contrast, with proximity scoring enabled, a total of 24 alleles were misassigned using ONT data, of which 22 were reassigned correctly when realigning the consensus sequences back to the MLST schemes (see Fig. 1). This led to an accuracy of 99.80% and 97.59% with and without reassignment of imperfect matches, respectively. The need for reassignment gradually decreased with increasing sequencing depth, where only three alleles needed to be reassigned at depths above 75 (see Fig. 1).

The two alleles that were not reassigned correctly with ONT reads belonged to the same low-depth *Staphylococcus aureus* isolate sequenced using an r9.4.1 flow cell, where one allele (*glpF_8* misassigned as *glpF_761*) was sequenced to a depth of coverage of 6.0, while the other (arcC_10 misassigned as arcC_755) was sequenced to a depth of coverage of 24.74 while being misassigned due to a homopolymer deletion (seven A’s predicted as six A’s). The *S. aureus* sample failed the internal quality control of SSI as well, containing only 8531 reads (52.9 Mbp) compared with [46050; 245325] reads ([445.4; 1223.5] Mbp) for the remaining *S. aureus* isolates of the same sequencing run. Such isolates would have been subjected to re-sequencing in usual cases when Illumina sequencing had not been performed simultaneously, which would most likely result in a correct typing of the *S. aureus* isolate. However, closer examination of the *arcC_755* revealed that the allele originated from a 454-sequenced isolate, which historically has been known for homopolymer errors like the ones known from ONT [56, 57]. Furthermore, the allele has only been identified once since its inclusion in 2011 [38]. The curators of the *S. aureus* MLST scheme have been informed of the findings, who confirm this to be a likely artifact of a sequencing error. Removing *arcC_755* from the *S. aureus* scheme caused the reads to be properly assigned to *arcC_10*, with the homopolymeric region being correctly polished to match the reference. The fact that we were able to detect this database artifact and adjust our findings accordingly highlights the utility of using MLST as an evaluation system.

Krocus misassigned eight alleles, giving an accuracy of 99.19%. The misassigned alleles could not be examined further, as Krocus is solely mapping-based and thus is not able to provide information on how the alleles were different.

**Figure 2.**
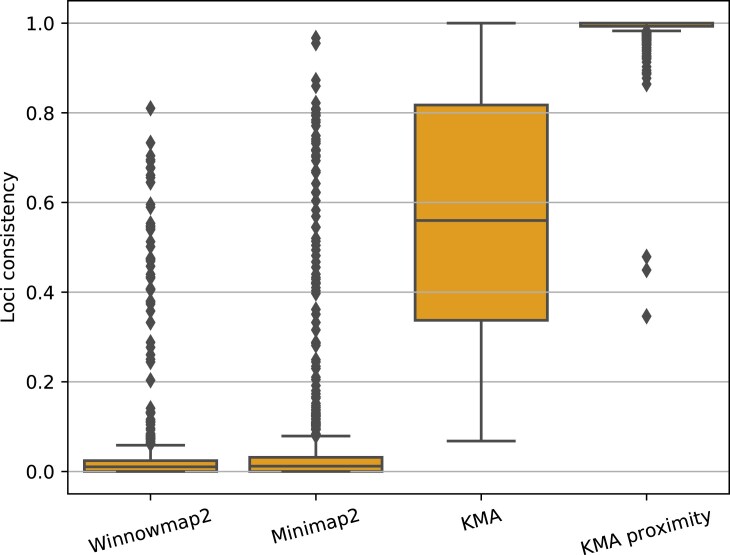
Consistency of assigned allele on each locus. Consistency was measured as the depth of coverage of the predicted allele on each locus, divided by the total depth of each locus. Krocus was excluded, as it only outputs one allele per locus, for which certainty is not provided.

The consistency of assigned alleles on each locus was measured as the depth of coverage of the predicted allele on each loci, divided by the total depth of each locus. When analyzing ONT data with KMA without proximity scoring, the average consistency was 56%, with consistencies starting from 6.8% (see Fig. 2). With proximity scoring enabled, the average consistency of assigned alleles per loci was 99.4%, with the majority of the loci being above 98%. Three loci were detected with a consistency of 34.6%, 44.9%, and 47.9%, which all belonged to the *acsA* loci of *Pseudomonas aeruginosa*, where a homolog of the *acsA* enzyme was matched to an additional allele of that loci. These findings were consistent across sequence technologies, i.e. the same additional homologs were also found from raw Illumina reads as well as *de novo* assemblies.

Winnowmap2 and minimap2 had an average consistency of 3.6% and 5.5%, respectively. Winnowmap2 assigned 9.36% of the loci with a consistency > 5%, while minimap2 assigned 16.20% with a consistency >5%. (see Fig. 2).

### Computational requirements

The computational requirements were measured using GNU time on a MacBook Pro Mid 2015, with a 2.2 GHz Quad-Core Intel Core i7 processor and 16 GB 1600 MHz DDR3 RAM.

**Figure 3.**
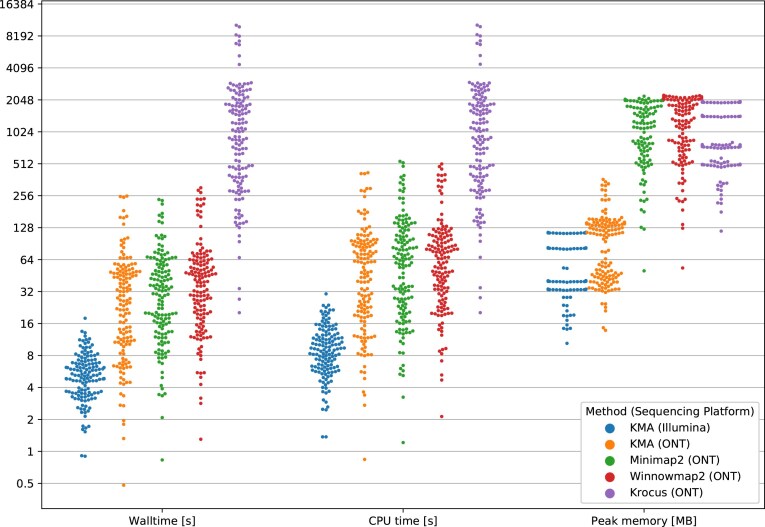
Computational requirements when performing MLST typing of Illumina and ONT data on a MacBook Pro Mid 2015.

With KMA, MLST typing of Illumina data required an average of 5.6 s, corresponding to 116 Mbp/s, with an average peak memory consumption of 71.2 MB, and these data were generally more computational efficient to analyze than ONT data, requiring 39.0 s, corresponding to 24 Mbp/s, with 106.0 MB peak memory consumption on average. Winnowmap2 and minimap2 performed similar to KMA, with an average of 54.30 and 42.80 s per sample, respectively. Krocus was more than an order of a magnitude slower, requiring 25.15 min per sample on average. Memory-wise, Winnowmap2, minimap2, and Krocus had similar average peak memory requirements of 1.38, 1.32, and 1.21 GB, respectively, placing them an order of magnitude above KMA (see Fig. 3).

## Discussion

In this work, we introduce and evaluate a new approach to perform MLST with low computational requirements, which is based on an assembly-free method that is computationally tractable with the resources of a conventional laptop computer.

We found that sequencing errors associated with the latest ONT flow cell technologies have minimal impact on MLST accuracy. The r10.3 flow cell chemistry is known for its higher accuracy but falls short of the r9.4.1 chemistry in terms of yield [58]. This tradeoff between accuracy and yield seems to be of minor concern in this study, as the higher quality reads revealed accurate assignments at the lower depths. With the r10.4.1 flow cell introduced by ONT, this tradeoff seems to be limited, as the higher accuracy has been maintained while increasing the yield to be comparable with that of the r9.4.1 flow cell [59]. As we expect the ONT technology to continue their development with decreased error rates, we tested the effect of proximity scoring on Illumina data as well. Setting the proxi option of KMA to −0.98 allowed a single error per Illumina read, assuming default alignment penalties and a read length of 150 bp, which correctly assigned the reads containing sequencing errors, giving slightly higher loci consistencies of the assigned alleles. However, due to the low error rate of Illumina, the typing results remained the same.

Multiplexing is a useful strategy to reduce the costs of sequencing; however, this practice introduces the risk of index hopping/swapping/jumping as seen earlier from Illumina data [6, 60]. With the majority of the loci consistencies for KMA lying above 98%, there is an indication of closely related low-depth alleles present in the samples. These low abundance matches support the findings of recent estimates of index hopping of ONT data, suggesting that these low abundance hits originate from samples sequenced simultaneously on the same flow cell [61].

Winnowmap2 and minimap2 were not built to resolve multimapping reads between references, but rather within references. The evaluation under this project showcases how random errors have the capability of matching actual differences between references, which makes it impossible to distinguish closely related alleles independently of the remaining reads. The inclusion of Winnowmap2 and minimap2 in this study should thus only be viewed as a baseline for the effect of having closely related sequences in one sequence database while treating individual query sequences independently. When aligning reads to a single reference genome, Winnowmap2 and minimap2 are likely more performant than KMA.

Although Krocus achieved a high accuracy, it was unable to provide information on imperfect matches, which hinders analysis of novel bacteria, as it will remain uncertain whether the isolate is truly novel or whether the result was due to low sequencing quality or other artifacts of the program. Moreover, the setup of Krocus is not transferable to other analysis problems, which ties it to MLST specifically.

The ability of KMA to type alleles directly from raw short- and long-read data using only a modern laptop avoids the need for large computational infrastructures. With the combination of ONT sequencing, this enables smaller laboratories to include sequencing as a routine tool, with limited startup costs. Computationally, the major bottleneck of the typing analysis is the initial base-calling of the ONT sequence data, which today can be carried out effectively on modern laptops [62]. With a peak memory below half a GB and a mean runtime around a minute, efficient typing can be achieved with KMA, without access to high-performance computing, as these analyses can essentially be accomplished with the capacity found on most smartphones today.

The solution provided here will allow for a larger inclusion of low- and middle-income countries in the era of sequencing, which will aid the global microbial surveillance of known pathogens and their epidemiology.

## Supplementary Material

lqaf116_Supplemental_Files

## Data Availability

Bitbucket: https://bitbucket.org/genomicepidemiology/kma Bioconda: https://bioconda.github.io/recipes/kma/README.html Operating system(s): UNIX OS. Programming language: C. Other requirements: zlib development files. License: Apache v2.0. Commands to reproduce results: S1.txt Sequencing data: S2.xlsx
